# Biomechanical analysis of stress around the tilted implants with different cantilever lengths in all-on-4 concept

**DOI:** 10.1186/s12903-022-02520-8

**Published:** 2022-11-05

**Authors:** Qi Wang, Zhen-zhen Zhang, Shi-zhu Bai, Shao-feng Zhang

**Affiliations:** 1 State Key Laboratory of Military Stomatology & National Clinical Research Center for Oral Diseases & Shaanxi Key Laboratory of Stomatology, Department of Pediatric Dentistry, School of Stomatology, The Fourth Military Medical University, No.145, Changle West Road, Xincheng District, Shaanxi Xi’an, China; 2 State Key Laboratory of Military Stomatology & National Clinical Research Center for Oral Diseases & Shaanxi Key Laboratory of Oral Diseases, Department of Prosthodontics, School of Stomatology, The Fourth Military Medical University, No.145, Changle West Road, Xincheng District, Shaanxi Xi’an, China

**Keywords:** All-on-4, Tilted implants, CAD, RP, Photoelastic stress analysis

## Abstract

**Background:**

Many clinical studies have reported the high success rate of the All-on-4 concept. In the present study, we aimed to compare the stress distribution with different tilted distal implants and cantilever lengths in an All-on-4 system using the two-dimensional photoelastic method and to establish the All-on-4 implant photoelastic model by computer-aided design (CAD) and rapid prototyping (RP).

**Methods:**

The data of the human edentulous mandible were acquired by computed tomography (CT). Three human edentulous mandible All-on-4 implant models with different distally inclined implant holes were fabricated using Mimic, Geomagic Studio software, and a light solidifying fast shaping machine. Then the final photoelastic models were established through the traditional method. Each of the three models had four NobelSpeedy Replace implants between the interforaminal regions. The two posterior implants were placed 0, 15, and 45 degrees distally before the mental foramen. The four implants were splinted by wrought cobalt-chromium alloy frameworks. Each of the three photoelastic models was submitted to a 150 N vertical load at five points on the framework: the central fossa of the mandibular first molar, and 0 mm, 5 mm, 10 mm, and 15 mm of the cantilever length. The stress produced in the models was photographed with a digital camera, and the highest value of the stressed fringe pattern was recorded.

**Results:**

The All-on-4 implant photoelastic model established by CAD and RP was highly controllable and easy to modify. The position and inclination of implants were accurate, and the frameworks could be passively emplaced. The stress values were higher around a single tilted implant compared with the distal implant in All-on-4 with the same inclination. The 0-degree distal implant and 45-degree distal implant demonstrated the highest and lowest stress when loading at the central fossa of the mandibular first molar, respectively. With the same inclination of distal implant, the peri-implant bone stress increased as the length of cantilever increased.

**Conclusion:**

The method of establishing the All-on-4 implant photoelastic model by CAD and RP was highly controllable, convenient, fast, and accurate. The tilted implants splinted in the fully fixed prosthesis with reduced cantilever lengths did not increase the stress level compared with the vertical distal implants.And this illustrated that the influence of cantilever on stress distribution was greater than the influence of implant inlination.

**Supplementary Information:**

The online version contains supplementary material available at 10.1186/s12903-022-02520-8.

## Background

The full-arch fixed prostheses (FFPs) supported by implants have been reported with a high success rate and patient satisfaction [1–5]. However, the rehabilitation becomes rather complex for the atrophic edentulous mandible because of the anatomical structures in the posterior region, such as the maxillary sinus and mental foramen. The placement of implants between the mental foramen may result in a longer cantilever length of the framework, which increases the torque on the implants [6]. In these cases, other procedures have been proposed to overcome the limitations in the posterior maxilla or mandible. Short implants as the least intrusive treatment are indicated [7, 8]. When the bone height is insufficient even for short implants, other procedures may be used, including bone grafting and zygomatic implants. However, both the above-mentioned treatments have limitations, such as surgical risk and complexity, and longer rehabilitation after the operation, which decreases the patient’s acceptance [9–11]. The use of tilted implants as an alternative has been proposed for the atrophic edentulous mandible. Malo et al. have proposed an All-on-4 concept in 2003 and 2005 for the treatment of edentulous mandible and maxillary, which can rehabilitate the edentulous jaws with four implants, including two vertical implants parallel to each other in the anterior region and two distally tilted implants in the posterior region. Tilted implants not only allow the placement of longer implants in the posterior region, avoid the bone graft, but also eliminate or reduce the cantilever extensions. Chrcanovic et al. have reported that differently angled implants may not affect the survival rate of implants and the loss of marginal bone [12]. Several studies have reported that tilted implants with shorter cantilever lengths do not increase the stress around the implants in the All-on-4 concept [13, 14]. Studies reported that for FFPs supported by four to six implants, the maximum cantilever length should be no more than twice the anterior/posterior (AP) length. And other researchers have recommended that full-arch, screw-retained, hybrid prostheses with CL/AP ratios less than 1.0 resulted in virtually complication-free prostheses [15, 16]. Nevertheless, few studies have reported the effect of cantilever length on stress distribution in All-on-4.

Photoelastic stress analysis has been widely applied to study dental biomechanics, which was first appeared in dental research with the publication by Noonan [17]. Over the years, the conventional method to establish the photoelastic model is a complex and time-consuming process, there may be some errors for the establishment of an implant photoelastic model, and the angle and site of the implants can not be accurately controlled. With the development of computer technology, computer-aided design (CAD)/computer-aided manufacturing (CAM) in combination with rapid prototyping (RP) has been widely used to design and fabricate the fixed, removable prostheses, implant surgical templates, and maxillofacial prostheses, which have been proved to be more precise and rapid [18, 19]. However, there are few studies about whether it can be used to fabricate the implant photoelastic model. Therefore, we aimed to explore the feasibility of the method to build the edentulous mandible All-on-4 implant photoelastic model with various distally tilted implants by CAD and RP technics and to evaluate the stress distribution at the bone-implant interface of tilted implants in an All-on-4 configuration with different inclinations of distal implants and cantilever lengths using photoelastic stress analysis.

## Methods

### Statement

The materials used in the research were acrylic resin, cobalt-chromium alloy and implant. And no experiments on humans were performed in this experiment /or no human tissue samples were used, therefore ethics were not involved in the study.

### Construction of photoelastic models

**Fig. 1:**
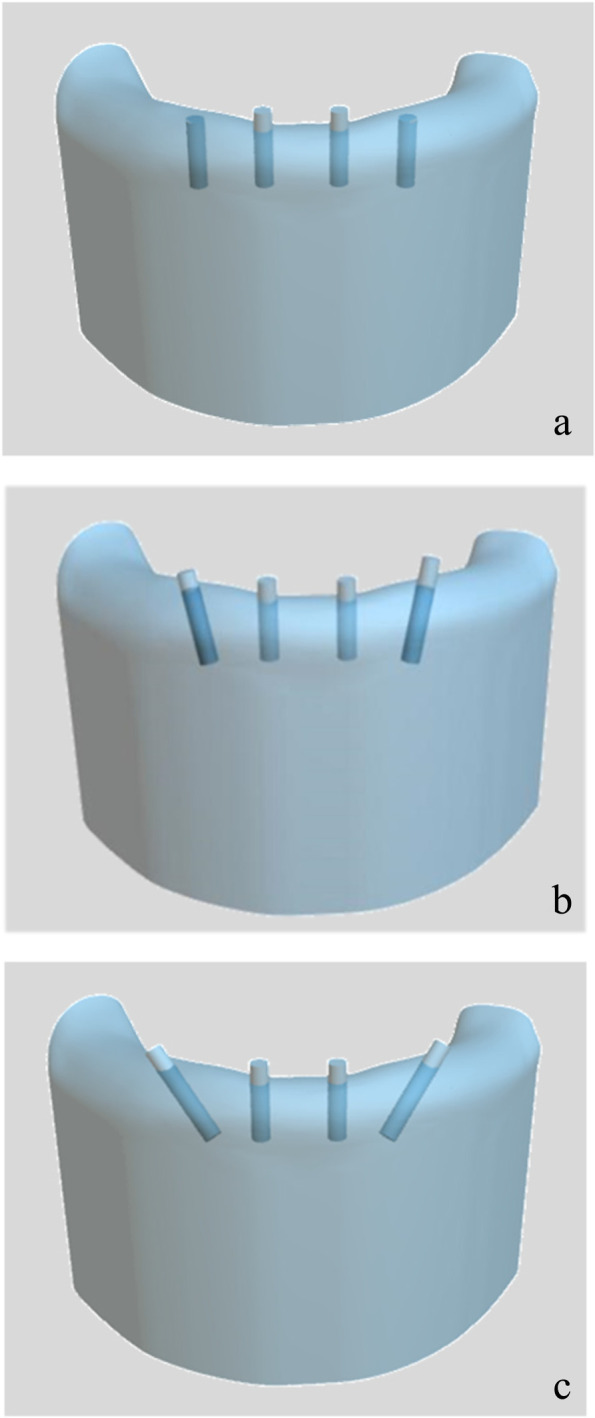
Three-dimensional mandibular model with four cylinders simulating implants. (**a**) 0-degree distal implant model; (**b**)15-degree distal implant model; (**c**) 45-degree distal implant model

**Fig. 2:**
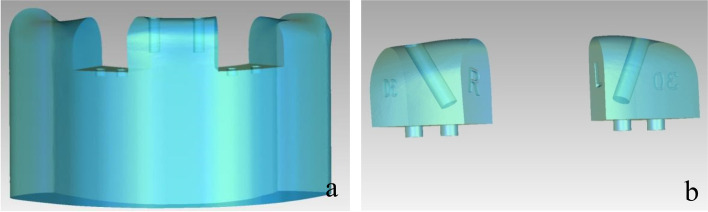
Modular models. (**a**) Base module; (**b**) two smaller modules

The edentulous mandible data were acquired by computed tomography, and then the data were imported into Mimcs10.0 software to reconstruct a three dimensional (3D) model of the edentulous mandible. Geomagic Studio 13.0 software was used to edit and fair the surface of the edentulous mandible 3D models. The three models were then heightened to 35 mm considering the influence of counterforce. The anterior region was 12 mm wide, and the posterior area was 15 mm wide. In all models, two cylinders were designed to simulate implants in the lateral incisor region, which were perpendicularly aligned to the eventual occlusal plane with an interimplant distance of 12 mm. The two distal cylinders were placed 6 mm anterior to the mental foramen. In the first model, the two distal cylinders were perpendicularly placed to the occlusal plane with the AP spread of 4.8 m. In the other two models, the two distal cylinders were distally tilted with an angle of 15 and 45 degrees respectively following the All-on-4 arrangement and the AP spread were 5.7 and 9.3 mm respectively. All cylinders were 12 mm long and 2.5 mm wide (Fig. 1a, b and c). The implant sites were reserved in the mandible models with the Boolean operation by subtracting the cylinder models and mandible models. Subsequently, each of the three models was designed into three modular structures according to the position of the two distal cylinders, in which the model was cut off at 2 mm anterior and posterior to the distal implants to acquire a larger mandibular base module and two smaller modules including the tilted cylinders (Fig. 2a and b). Two cylindrical tenon structures (2.0 × 3.0 mm) were designed between the base module and small modules. Once the desired mandible module models were obtained, they were saved as a file of standard STL format with the Geomagic Studio 10.0 software. The file of STL format was transmitted into stereolithography (SLA) machine to convert the 3D image data into the thin section data. Moreover, the resin models were finally obtained using SLA technology. Three resin combination models with different distally tilted implants could be obtained by combining the one base module and six small modules.

Three photoelastic models with implant sites were constructed with photoelastic acrylic resin by the conventional mold-and-pour acrylic resin technique. The Nobel planting system was used to expand the implant hole in the photoelastic models step by step according to the position and direction. Then all implants (4.0 × 13 mm, Speedy Replace, Nobel Biocare) were embedded into the mandible photoelastic models. In addition, 4-mm multiunit abutments (Nobel Biocare) were connected to the axial implants, 4-mm, 17-degree multiunit abutments were connected to the 15-degree tilted implants, and 30-degree multiunit abutments were connected to the 45-degree tilted implants and tightened to 30 Ncm using a manual torque wrench.

### Fabrication of cobalt-chromium alloy framework

**Fig. 3:**
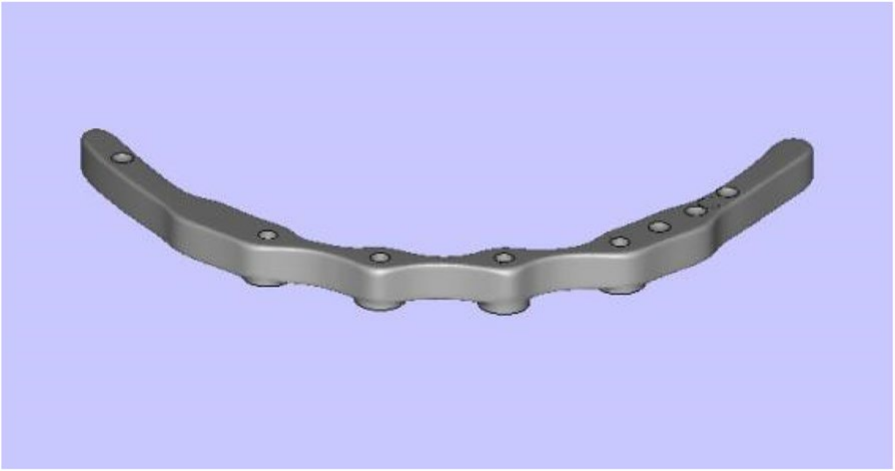
Three-dimensional framework model

**Fig. 4:**
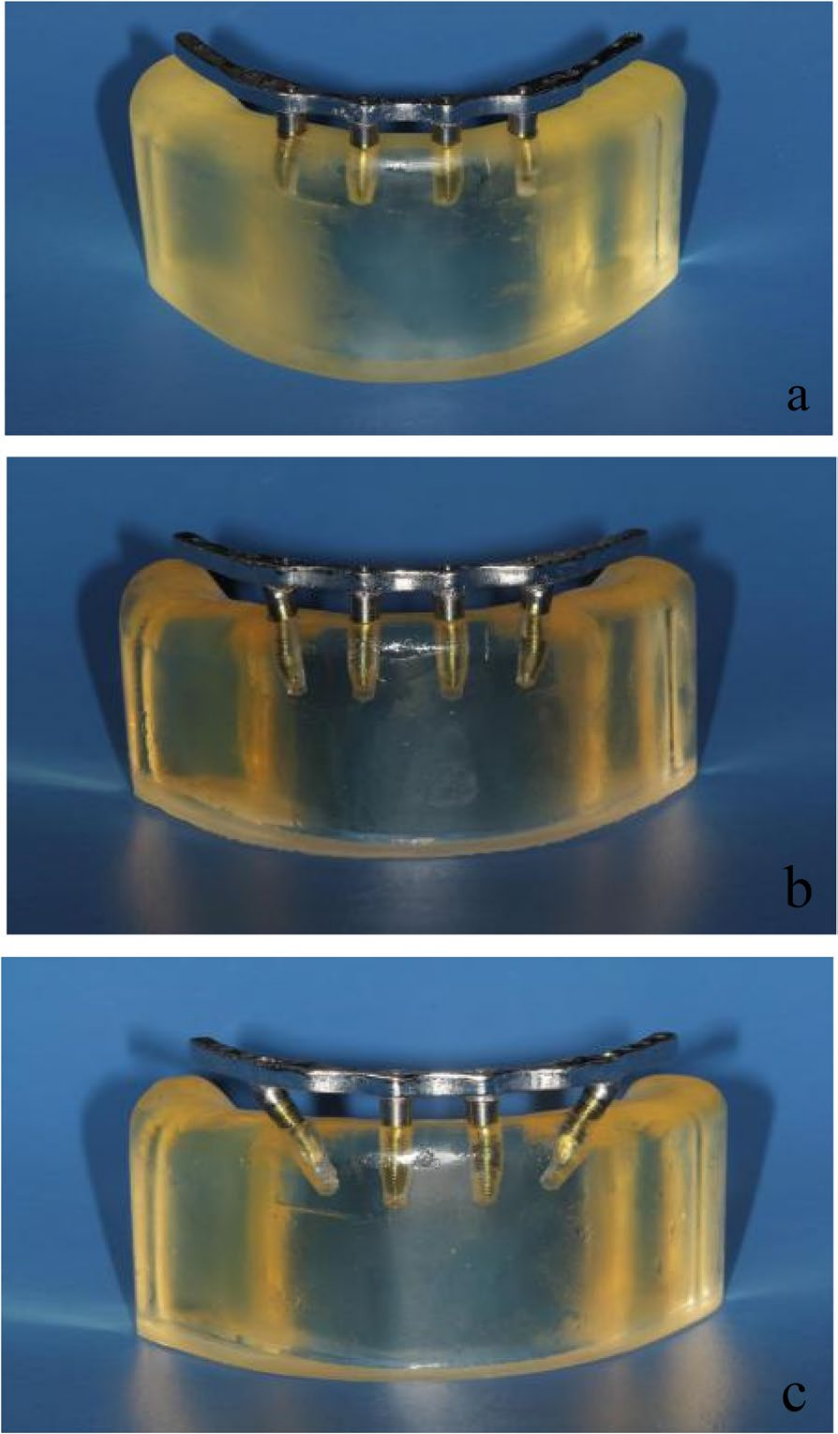
All-on-4 implant photoelastic models. (**a**) 0-degree distal implant of All-on-4 model; (**b**) 15-degree distal implant of All-on-4 model; (**c**) 45-degree distal implant of All-on-4 model

The framework models were designed with the Geomagic Studio 10.0 software according to the morphology of the mandibular alveolar ridge with a height of 4 mm, and the models were spaced out the mandibular alveolar ridge 4 mm apart. For each of the framework models, two cone structures were designed to imitate the superstructure of the multiunit abutments and two of 17- and 30-degree multiunit abutments respectively. The internal structure and the height of the cone structures were equal to the superstructure of the multiunit abutments. The frameworks which could closely combined with multiunit abutments were obtained through the Boolean operation by subtracting the cone structures and the framework model, the arrangements of which were the same as the implants in the mandible models. For the application of the load, five disc-shaped structures were built on the upper surface of the framework at a cantilever length of 0 mm, 5 mm, 10 mm, and 15 mm, and the central fossa of the mandibular first molar. Then the framework models were saved as a file of standard STL format (Fig. 3), and the Co-Cr alloy frameworks were fabricated. Multiunit abutments were fixed to the frameworks by 20 Ncm torque screws with a manual torque wrench (Fig. 4a, b and c).

### Photoelastic stress analysis

**Fig. 5:**
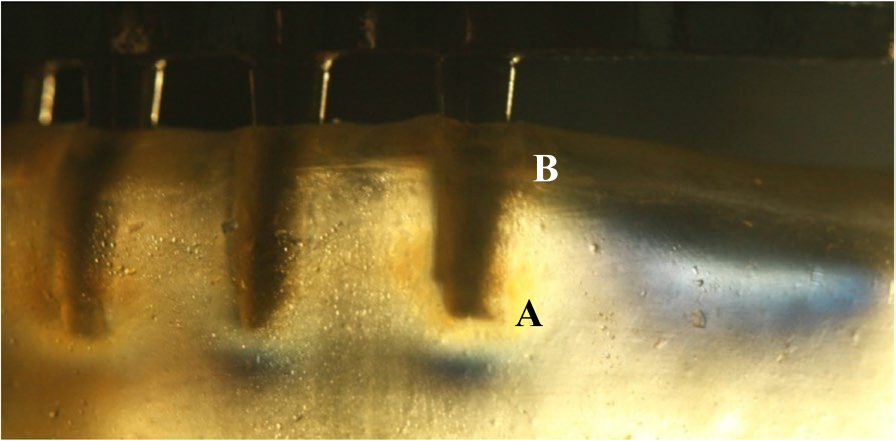
Stresses in unloaded All-on-4 implant photoelastic model

In the first test (test 1), each of the photoelastic models was placed on an anvil before and after the frameworks were tightened and the distal implant received vertical static load of 200 N. In the second test (test 2), a vertical load of 150 N was applied to the framework representing the central fossa of the mandibular first molar of each model. The cantilever lengths in the 0-,15- and 45-degree model were 20.4 mm, 18.2 mm and 9.3 mm. Then a vertical load of 150 N was applied to the framework of the three photoelastic models at four loading points: cantilever length of 0mm, 5 mm, 10 mm, and 15 mm. Isochromatic fringes produced in the acrylic resin models at the time of loading were photographed with a digital camera (Canon). The images obtained in the field of the circular polariscope were used to qualitatively analyze the stress distribution. Two sides of the models were recorded separately: left and right. The fringes were analyzed for two zones (apical:A and distal crest:B) as shown in Fig. 5.

The isochromatic fringes observed in a polariscope were generated from a passage of polarized light passing through a photoelastic model. A stress-free condition that was observed in the circular polariscope appeared black. When the load was applied to the model, a series of consecutive bands with different colors appeared in the stressed region-grey, white, pale yellow, orange, dull red, purple, deep blue, blue-green, yellow, orange, rose red, purple, green, yellow, red, and green. The dividing zone between red and blue was the first order, and that between the red and green was the second order [20]. Beyond this point, once a repetition of pink and green colors was observed, the transition indicated a new fringe order. Orders could be assigned to the next fringe, and when one fringe was identified, we should make sure that the direction of increasing fringe order corresponded to the correct color sequence. The number of fringes provided information on the strain magnitude. The region where fringes were closer to each other indicated higher stress concentrations.

## Results

The All-on-4 implant photoelastic model was close to 1:1 with the original model. Moreover, the model was featured a faintly yellowish, bright surface, homogeneous structure, and high optical sensitivity. The position and inclination of implants were accurate, and the frameworks could be passively emplaced. The model was observed without initial stress. The stress distribution of the implant-bone interface with tilted distal implants and cantilever lengths could be qualitatively analyzed.

**Fig. 6:**
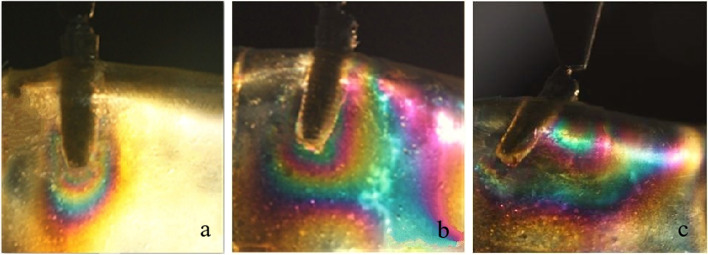
Stress distribution around single implants under a 200 N load. (**a**) Axial implant; (**b**) 15 degrees tilted implant; (**c**) 45 degrees tilted implant

**Fig. 7:**
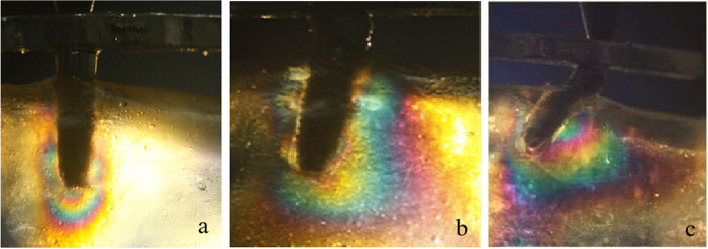
Stress distribution around the distal implants of the All-on-4 photoelastic models under a 200 N load. (**a**) Axial model; (**b**) 15 degrees tilted model; (**c**) 45 degrees tilted model

In test 1, single implant and All-on-4 distal implant showed similar isochromatic fringe patterns and fringe orders. The stresses were observed both in the distal crest and apical area of the tilted implants. However, for the vertical implant, the fringe patterns were mainly concentrated in the apical area. The photoelastic models and implant distribution was symmetrical on both side of the model, so when loading on the right and left side respectively the fringe patterns around the distal implant were similar on each side. Therefore, only the results of the left side were presented. The stress at the distal crest of the implant was increased as the inclination of the implant was increased. However, the stress in the apex of the implant was decreased. Figure 6a, b and c shows the isochromatic fringe patterns around the single implants. Figure 7a, b and c represents the stress distribution in the per-implant bone around the implants of the All-on-4 photoelastic models.

For the axial implant, the 200 N load on the implant generated 2.65 and 1.0 fringe order of stress at the apex and neck of the implant, respectively. A 1.39 fringe order was observed at the distal crest, and a 2.35 fringe order of stress was observed in the apex of the 15-degree tilted implant. Loading on the framework of the 15-degree tilted implant resulted in a 1.22 fringe order of stress at the distal crest and a 1.82 fringe order of stress at the apex of the inclined implant. The maximum fringe order of 3 developed in the crest around the single 45-degree tilted implant and a 2.35 fringe order in the apex. For the 45-degree tilted implant of the All-on-4 model, a 2.65 fringe order was observed at the distal crest, and a 2 fringe order of stress was observed in the apex.

**Fig. 8:**
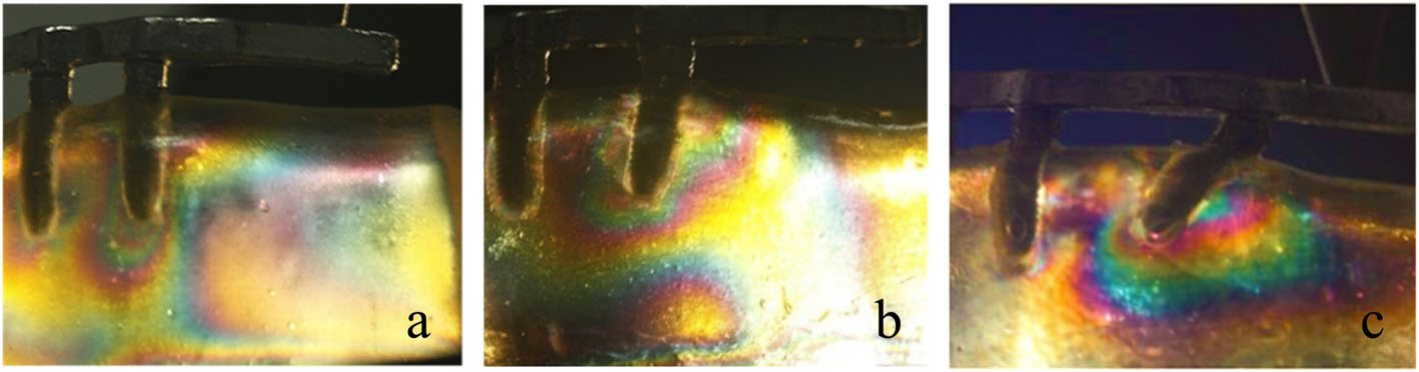
Stress distribution around the distal implants when loading at the first molar. (**a**) Axial model; (**b**) 15 degrees tilted model; (**c**) 45 degrees tilted model

**Fig. 9:**
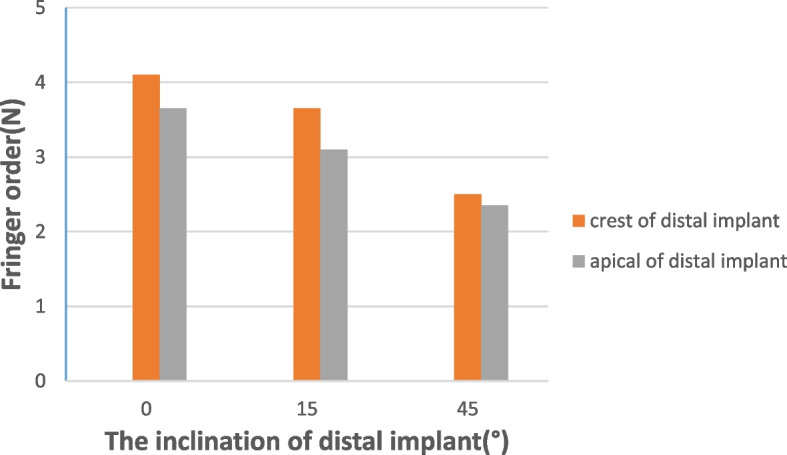
Fringe order around the distal implants when loading at the first molar

**Fig. 10:**
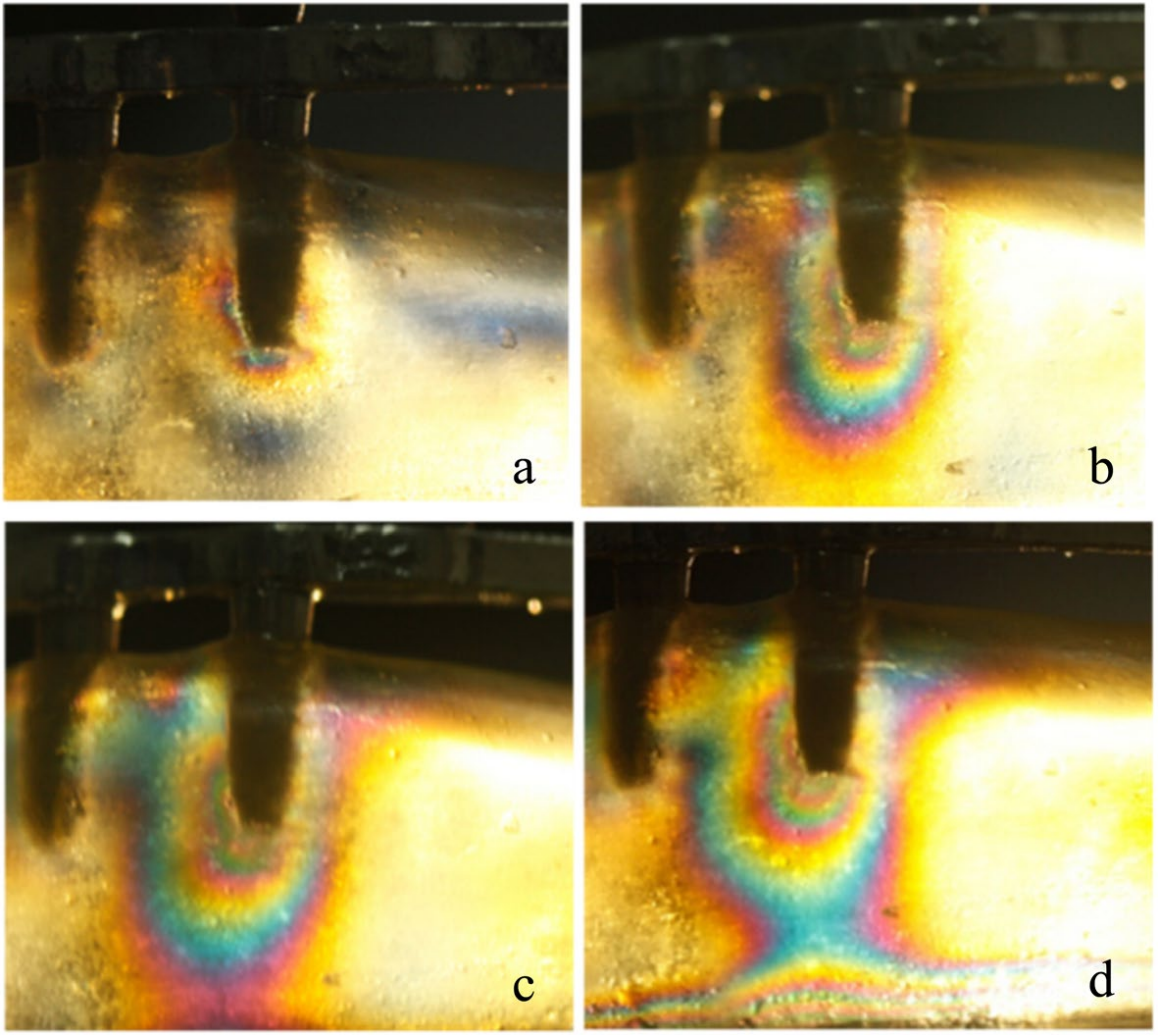
Stress distribution around the 0-degree distal implants with different cantilever length under a 150 N load. (**a**) 0 mm of cantilever length; (**b**) 5 mm of cantilever length; (**c**) 10 mm of cantilever lenght; (**d**) 15 mm of cantilever length

**Fig. 11:**
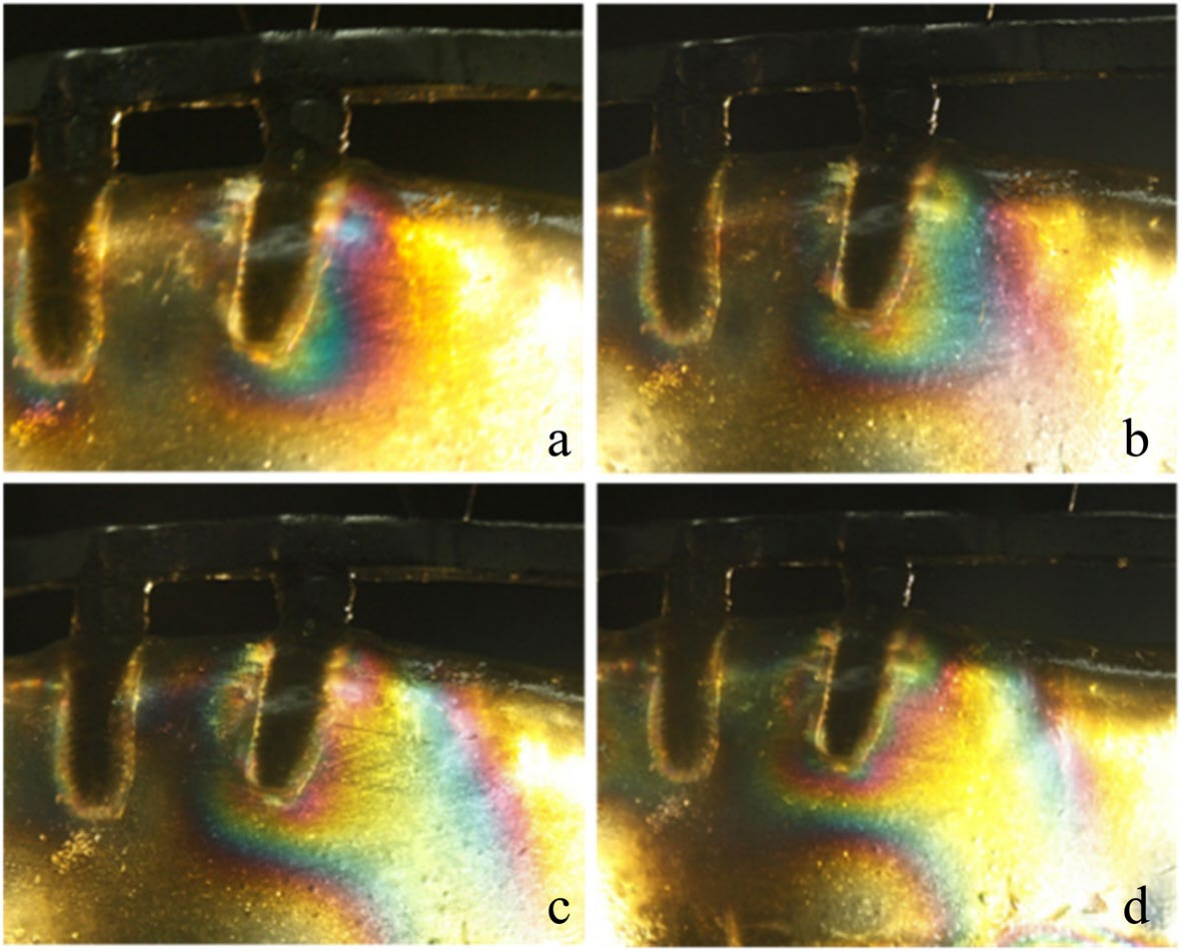
Stress distribution around the 15-degree distal implants with different cantilever length under a 150 N load. (**a**) 0 mm of cantilever length; (**b**) 5 mm of cantilever length; (**c**) 10 mm of cantilever lenght; (d) 15 mm of cantilever length

**Fig. 12:**
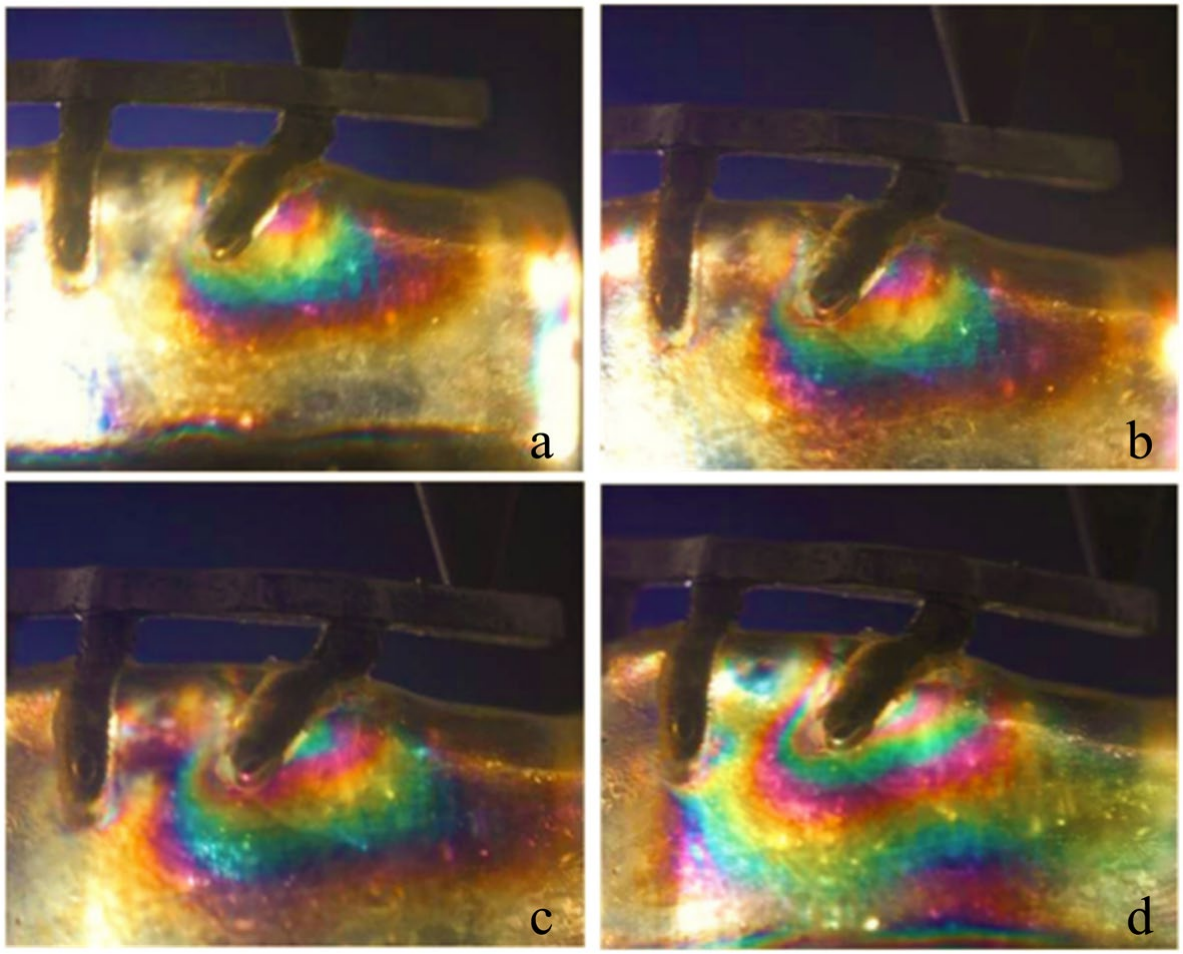
Stress distribution around the 45-degree distal implants with different cantilever length under a 150 N load. (**a**) 0 mm of cantilever length; (**b**) 5 mm of cantilever length; (**c**) 10 mm of cantilever lenght; (**d**) 15 mm of cantilever length

**Fig. 13:**
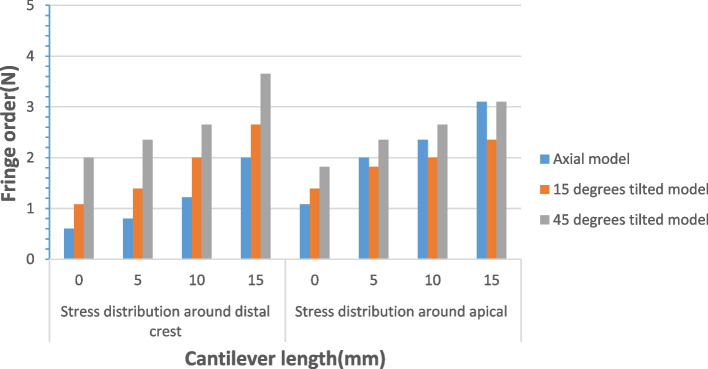
Fringe order around the distal implants with tilting distal implant and different cantilever length under a 150 N load

In test 2, when loading at the central fossa of the mandibular first molar, the use of a tilted implant reduced the cantilever length and the stress around the distal implant (Fig. 8a, b and c). Figure 9 illustrates the fringe orders observed in the peri-implant bone around the distal implants with graphic. The model with the maximum distal implant inclination (45-degree) corresponding to the minimum cantilever length (9.8 mm) produced the lowest 2.35 and 2.5 fringes of stresses at the apex and the distal neck of the tilted implant, respectively. Moreover, the model with four parallel implants placed in the interforaminal area with the maximum cantilever length (20.5 mm) generated the highest fringe order of 4.1 and 3.65 at the distal crest and the apex of the distal implant, respectively. In the three All-on-4 photoelastic models, the isochromatic fringe patterns were similar with the change of the cantilever length. The stress at the distal crest and apex of the distal implants was increased as the cantilever length was increased (Figs. 10, 11 and 12). Figure 13 provide graphic representations of the fringe orders of stress distribution surrounding the distal implant with different cantilever length. The model demonstrated that a 45-degree distal implant had higher peri-implant bone stress compared with the 0-degree tilted implant with the same cantilever length.

## Discussion

In the present study, we investigated the stress distribution at the neck and apex of the dental implants in the All-on-4 concept with varying inclinations of the distal implants and cantilever lengths, after the axial load in photoelastic models mimicking an edentulous mandible. The photoelasticity technique has the advantage of intuitive view and accuracy, which allows the observation of stress throughout the photoelastic model and the location of the concentrations of stress. However, fabricating the photoelastic model is a much complex and time-consuming process, and has low accuracy. Therefore, CAD/RP was used to build the edentulous mandible All-on-4 implant photoelastic models in this study. The implant sites were reserved in the 3D mandibular and the loading sites on the 3D framework were also reserved, so that we can control the implants position, inclination and loading accurately. The 3D edentulous mandible model was divided into the base module and two small modules including the tilted implants. And then the 3D models were constructed into resin models under the computer control, so the defects of the models could be found and modified in time. This ensured that the position of two vertical implants in the anterior region remained constant when replacing the remote implant module, achieving a high consistency between groups. Modular methods can easily change the design according to different research needs. By replacing different planting modules to simulate different planting methods, the application is flexible and greatly saves the processing cost and time. This method not only simplified the procedure, saved the materials but also allowed to control the position and angle of the implant accurately and greatly reduced human error. However, in the present study, it was impossible to calculate the stress value of the measuring location in the 3D photoelastic models. Because of the different thicknesses and geometric configurations of the model, only a qualitative analysis could be made. Although using the fringe orders to represent the stresses around the implant might modify the magnitude of the stresses, the position and tendency of stress concentration would not be changed. Therefore, we were still able to effectively analyze the stress distribution around the distal implants of the All-on-4 implant photoelastic models.

Tilted implants are used as an alternative for the atrophic edentulous mandible to avoid anatomic obstacles, which allow using longer implants and shorter dental cantilever lengths. However, the peri-implant bone stresses around the single tilted implant have been reported to be two to five times higher compared with the stresses around the vertical implant [21]. In this study, Figs. 6 and 7 show the stress distribution around the single implant and the distal implants of the All-on-4 implant photoelastic models. It showed that tilted implants produced higher peri-implant bone stress compared with the vertical implants, and the stresses were increased when the inclination of the implant was increased. These results were consistent with previous finite element analyses that examine the load transmission using different implant inclinations [22, 23]. In addition, because of the distally tilted implants are splined with other three implants in a rigid metal framework, the rigidity of the framework and the anterior and posterior spread of the implants can reduce the stresses transmitted to the tilted implants [24, 25], so the stresses at the neck of the tilted implant of the All-on-4 implant photoelastic models are lower compared with the single implants of the same inclination. Balshi TJ et al. have reported that following the All-on-4 protocol, the cumulative implant survival rates of the tilted implants and axial implants are identical at 97.3% [26]. This finding indicates that tilted implants do not negatively affect the stress distribution at the peri-implant bone regions.

A finite element analysis has concluded that when submitted to a static load, the main stress concentration can be found in the cervical region of the implant [27]. However, in this study, when a vertical load was applied to the axial implant, no matter a solitary implant or the distal implant of All-on-4, it showed that the highest stress was concentrated at the apical region of the implant. The increased inclination of the distal implant resulted in decreased stress values in the apical region and increased stresses in the cervical region. A photoelastic study has demonstrated that when analyzing the stress distribution in the apical region, there are significant statistical differences between the inclinations, with the higher stresses for the implant with increasing angulation, which is consistent with the experimental results [28]. In addition, because the model cannot be constructed based on the elastic modulus of cortical bone and cancellous bone with the homogeneous and isotropic characteristics of the photoelastic model, the physical characteristics of the peri-implant tissues could be completely reproduced, which might result in the stress concentration in the apical region of the implant.

When the inclination of the distal implant remained constant, it seemed that the increase of cantilever length increased the cantilever length to AP spread ratio, and stress in the cervical and apical region of the implant was increased. However, with the same cantilever length, when the angulations of the distal implant were increased, the cantilever length to AP spread ratio was decreased. The stress value in the cervical region was increased, and the 45-degree model showed the highest values of stress. In the apical region, there was no obvious change. A retrospective study about the relationship between implant prosthetic complications in the patients with implant-fixed complete dental prosthesis and cantilever length to AP spread ratio has demonstrated that there is no significant effect on the complications no matter the AP spread ratio is greater than or less than or equal to 2.1 [29]. However, Carl Drago has stated that the cantilever length to AP spread ratio of interim, full-arch, screw-retained prostheses between 0.5 and 0.6 results in minimal prosthetic complications [30].

In the three All-on-4 implant photoelastic models, when loading on the central fossa of the mandibular first molar, the model of 0-degree and 45-degree distal implants showed the highest and lowest stress respectively. Which indicating that compared with the inclination of the distal implant, the change of the stress distribution of the peri-implant tissues was more significant when the cantilever length was increased. Although the increase of the implant angle made the implant insertion more difficult, The All-on-4 concept was also a predictable treatment option for patients with a severely atrophic mandible because of its better stress distribution and lower expenses.

## Conclusion

Within the limitations of this photoelastic stress analysis, it is simple and accurate to establish the All-on-4 implant photoelastic model by CAD and RP, which can easily control the differences between model groups and analyze the stress around the implant-bone interface. Single tilted implant and distal implant of the All-on-4 models increased the stress around the implant compared with the vertical implant. Moreover, the stress around the distal implant of the All-on-4 models was lower compared with a single implant with the same inclination of the implant. In case that the implant cannot be implanted vertically in the posterior region of the atrophic edentulous mandible, the use of tilted distal implants in the All-on-4 fixed prostheses not only shortened the surgical period, but also reduced the cantilever length, decreased the maximum peri-implant bone stresses and improved the survival rate of the implants and restoration. The cantilever length of the All-on-4 fixed prostheses played a key role in the reduction of the stresses around the implants.

## Supplementary Information

**Additional file 1.**

## Data Availability

The authors confirm that the data generated or analysed during this study are included in this published article [and its supplementary information file].

## Abbreviations

CAD: Computer Aided Design
RP: Rapid Prototyping
CT: Computed tomography
FFPs: The full-arch fixed prostheses
AP: Anteroposterior
SLA: Stereolithography

## References

[1] Barootchi S, Askar H, Ravidà A, Gargallo-Albiol J, Travan S, Wang H-L (2020). Long-term clinical outcomes and cost-efectiveness of full-arch implant-supported zirconia-based and metal-acrylic fxed dental prostheses: a retrospective analysis. Int J Oral Maxillofac Implants..

[2] de Luna Gomes JM, Lemos CAA, Santiago Junior JF, de Moraes SLD, Goiato MC, Pellizzer EP (2019). Optimal number of implants for complete-arch implant-supported prostheses with a follow-up of at least 5 years: A systematic review and meta-analysis. J Prosthet Dent..

[3] Agliardi E, Panigatti S, Clericò M, Villa C, Malò P (2010). Immediate rehabilitation of the edentulous jaws with full fixed prostheses supported by four implants:interim results of a single cohort prospective study. Clin Oral Implants Res..

[4] Mohamed LA, Khamis MM, El-Sharkawy AM, Fahmy RA (2021). Evaluation of immediately loaded mandibular four vertical versus tilted posterior implants supporting fixed detachable restorations without versus with posterior cantilevers. Oral Maxillofac Surg.

[5] Maló P, de Araújo Nobre M, Lopes A, Ferro A, Botto J (2019). The All-on-4 treatment concept for the rehabilitation of the completely edentulous mandible: A longitudinal study with 10 to 18 years of follow-up. Clin Implant Dent Relat Res..

[6] Ozan Oguz, Kurtulmus-Yilmaz Sevcan (2018). Biomechanical Comparison of Different Implant Inclinations and Cantilever Lengths in All-on-4 Treatment Concept by Three-Dimensional Finite Element Analysis. Int J Oral Maxillofac Implants..

[7] Kim SY, Ku JK, Kim HS, Yun PY, Kim YK (2018). A retrospective clinical study of single short implants (less than 8 mm) in posterior edentulous areas. J Adv Prosthodont..

[8] Coskunses FM, Tak Ö (2021). Clinical performance of narrow-diameter titanium-zirconium implants in immediately loaded fixed full-arch prostheses: a 2-year clinical study. Int J Implant Dent..

[9] Ramezanzade S, Yates J, Tuminelli FJ, Keyhan SO, Yousefi P, Lopez-Lopez J (2021). Zygomatic implants placed in atrophic maxilla: an overview of current systematic reviews and meta-analysis. Maxillofac Plast Reconstr Surg..

[10] Esposito M, Grusovin MG, Felice P, Karatzopoulos G, Worthington HV, Coulthard P (2009). The efficacy of horizontal and vertical bone augmentation procedures for dental implants - a Cochrane systematic review. Eur J Oral Implantol..

[11] Jensen OT, Adams MW (2014). Anterior sinus grafts for angled implant placement for severe maxillary atrophy as an alternative to zygomatic implants for full arch fixed restoration: technique and report of 5 cases. J Oral Maxillofac Surg.

[12] Chrcanovic BR, Albrektsson T, Wennerberg A (2014). Tilted versus axially placed dental implants: A meta-analysis. J Dent..

[13] Saleh Saber F, Ghasemi S, Koodaryan R, Babaloo A, Abolfazli N (2015). The Comparison of Stress Distribution with Different Implant Numbers and Inclination Angles In All-on-four and Conventional Methods in Maxilla: A Finite Element Analysis. J Dent Res Dent Clin Dent Prospects..

[14] Ozan O, Kurtulmus-Yilmaz S (2018). Biomechanical Comparison of Different Implant Inclinations and Cantilever Lengths in All-on-4 Treatment Concept by Three-Dimensional Finite Element. Int J Oral Maxillofac Implants..

[15] Sertgöz A, Güvener S (1996). Finite element analysis of the effect of cantilever and implant length on stress distribution in an implant-supported fixed prosthesis. J Prosthet Dent..

[16] Drago Carl (2018). Ratios of Cantilever Lengths and Anterior-Posterior Spreads of Definitive Hybrid Full-Arch, Screw-Retained Prostheses: Results of a Clinical Study. J Prosthodont..

[17] Farah JW, Craig RG, Sikarskie DL (1973). Photoelastic and finite element stress analysis of a restored axisymmetric first molar. J Biomech.

[18] Nayar S, Bhuminathan S, Bhat WM (2015). Rapid prototyping and stereolithography in dentistry. J Pharm Bioallied Sci..

[19] Harris BT, Montero D, Grant GT, Morton D, Llop DR, Lin WS (2017). Creation of a 3-dimensional virtual dental patient for computer-guided surgery and CAD-CAM interim complete removable and fixed dental prostheses: A clinical report. J Prosthet Dent..

[20] Kim KS, Kim YL, Bae JM, Cho HW (2011). Biomechanical Comparison of Axial and Tilted Implants for Mandibular Full-Arch Fixed Prostheses. Int J Oral Maxillofac Implants.

[21] Cehreli M, Duyck J, De Cooman M, Puers R, Naert I (2004). Implant design and interface force transfer: A photoelastic and strain-gauge analysis. Clin Oral Implants Res.

[22] Zampelis A, Rangert B, Heijl L (2007). Tilting of splinted implants for improved prosthodontic support: A two-dimensional finite element analysis. J Prosthet Dent.

[23] Kumari A, Malhotra P, Phogat S, Yadav B, Yadav J, Phukela SS (2020). A finite element analysis to study the stress distribution on distal implants in an all-on-four situation in atrophic maxilla as affected by the tilt of the implants and varying cantilever lengths. J Indian Prosthodont Soc..

[24] Bevilacqua M, Tealdo T, Pera F, Mossolov A, Drago C, Pera P (2008). Three-dimensional finite element analysis of load transmission using different implant inclinations and cantilever lengths. Int J Prosthodont.

[25] Menini M, Pesce P, Bevilacqua M, Pera F, Tealdo T, Barberis F, Pera P (2015). Effect of Framework in an Implant-Supported Full-Arch Fixed Prosthesis: 3D Finite Element Analysis. Int J Prosthodont..

[26] Balshi TJ, Wolfinger GJ, Slauch RW, Balshi SF (2014). A retrospective analysis of 800 Brånemark System implants following the All-on-Four™ protocol. J Prosthodont..

[27] Nokar S, Jalali H, Nozari F, Arshad M (2020). Finite Element Analysis of Stress in Bone and Abutment-Implant Interface under Static and Cyclic Loadings. Front Dent..

[28] Begg T, Geerts GA, Gryzagoridis J (2009). Stress patterns around distal angled implants in the all-on-four concept configuration. Int J Oral Maxillofac Implants.

[29] Purcell BA, McGlumphy EA, Yilmaz B, Holloway JA, Beck FM (2015). Anteroposterior Spread and Cantilever Length in Mandibular Metal-Resin Implant-Fixed Complete Dental Prostheses: A 7- to 9-Year Analysis. Int J Prosthodont..

[30] Drago C (2017). Cantilever Lengths and Anterior-Posterior Spreads of Interim, Acrylic Resin, Full-Arch Screw-Retained Prostheses and Their Relationship to Prosthetic Complications. J Prosthodon.

