# Supplementary material for: Biomechanical analysis of stress around the tilted implants with different cantilever lengths in all-on-4 concept
Source: BMC Oral Health. 2022 Nov 5;22:469. doi: 10.1186/s12903-022-02520-8 (PMC9636816; doi:10.1186/s12903-022-02520-8)
Supplement: Supplementary file 1 — Additional file 1. [file 12903_2022_2520_MOESM1_ESM.pdf]

The data generated or analysed during this study :

Text 1

Stress distribution around single implants and the distal implants of All-on-4

	The inclination of implant(°)		
	0	15	45
Single implant	2.65（apex）<br>1.0（neck）	2.35（apex）<br>1.39（neck）	2.35（apex）<br>3.0（neck）
distal implants of All-on-4	2.35（apex）<br>0.8（neck）	1.82（apex）<br>1.22（neck）	2.0（apex）<br>2.65（neck）

Text2

Fringe order around the distal implants when loading at the first molar

Inclination of distal implant(°)	Cantilever length(mm)	Fringe order	
		neck	apex
0	20.5	4.1	3.65
15	18.2	3.65	3.1
45	9.8	2.5	2.35

Fringe order around the distal implants with different cantilever length under a 150N load

Cantilever length（mm）	Inclination of distal implant(°)		
	0	15	45
0	1.08(apex)<br>0.6 (neck)	1.39(apex)<br>1.08 (neck)	1.82 (apex)<br>2.0 (neck)
5	2.0 (apex)<br>0.8 (neck)	1.82 (apex)<br>1.39 (neck)	2.35(apex)<br>2.35(neck)
10	2.35 (apex)<br>1.22 (neck)	2.0 (apex)<br>2.0 (neck)	2.65(apex)<br>2.65 (neck)
15	3.1 (apex)<br>2.0 (neck)	2.35 (apex)<br>2.65 (neck)	3.1 (apex)<br>3.65 (neck)
